# Modulating Frontal Networks’ Timing-Dependent-Like Plasticity With *Paired Associative Stimulation* Protocols: Recent Advances and Future Perspectives

**DOI:** 10.3389/fnhum.2021.658723

**Published:** 2021-04-22

**Authors:** Giacomo Guidali, Camilla Roncoroni, Nadia Bolognini

**Affiliations:** ^1^Neurophysiology Lab, IRCCS Istituto Centro San Giovanni di Dio Fatebenefratelli, Brescia, Italy; ^2^Department of Psychology, NeuroMI – Milan Center for Neuroscience, University of Milano-Bicocca, Milan, Italy; ^3^Laboratory of Neuropsychology, IRCCS Istituto Auxologico Italiano, Milan, Italy

**Keywords:** frontal cortex, *paired associative stimulation*, spike-timing-dependent plasticity, transcranial magnetic stimulation, executive functions, fronto-parietal (executive) network

## Abstract

Starting from the early 2000s, *paired associative stimulation* (PAS) protocols have been used in humans to study brain connectivity in motor and sensory networks by exploiting the intrinsic properties of timing-dependent cortical plasticity. In the last 10 years, PAS have also been developed to investigate the plastic properties of complex cerebral systems, such as the frontal ones, with promising results. In the present work, we review the most recent advances of this technique, focusing on protocols targeting frontal cortices to investigate connectivity and its plastic properties, subtending high-order cognitive functions like memory, decision-making, attentional, or emotional processing. Overall, current evidence reveals that PAS can be effectively used to assess, enhance or depress physiological connectivity within frontal networks in a timing-dependent way, in turn modulating cognitive processing in healthy and pathological conditions.

## Introduction

*Paired associative stimulation* (PAS) is a protocol of non-invasive brain stimulation in which a sensory, peripheral stimulus is repeatedly paired with a transcranial magnetic stimulation (TMS) pulse over a cortical area known to be activated by the former stimulus. By varying the inter-stimulus interval (ISI) between these stimulations, PAS protocols can affect synaptic plasticity, inducing long-term potentiation (LTP)-like and depression (LTD)-like after-effects on cortical excitability [i.e., *Spike-timing-dependent plasticity* (STDP); e.g., Caporale and Dan, 2008] in the stimulated cortical area or circuit (e.g., Stefan et al., 2000; Wolters et al., 2003). Over the last 2 decades, PAS literature moved from the widely replicated, classical protocol, pairing electric stimuli with M1-TMS (i.e., M1-PAS), to more complex protocols targeting sensory and crossmodal networks (e.g., Wolters et al., 2005; Schecklmann et al., 2011; Suppa et al., 2013, 2015; Sowman et al., 2014; Ranieri et al., 2019; Zazio et al., 2019; Guidali et al., 2020). Overall, these *peripheral-cortical* protocols proved to be robust and flexible tools to non-invasively investigate and interact with the plastic properties of sensorimotor networks in humans (for a review, see: Carson and Kennedy, 2013; Suppa et al., 2017). In the last 10 years, the *cortico-cortical* protocols (cc-PAS) have been developed, consisting of pairing a TMS pulse (in substitution to the sensory stimulus) with a pulse over a different – but interconnected – cortical area. The rationale behind them is using these protocols to target and influence the communication between two cortical nodes of a brain network. At variance with paired-pulse TMS, cc-PAS protocols can modulate the weight of the coupling between the two target areas, likely through the induction of associative plasticity (Koch, 2020). The first studies adopting the cc-PAS have focused on the motor system (e.g., Rizzo et al., 2009; Arai et al., 2011; Buch et al., 2011; Lu et al., 2012; Koch et al., 2013; Veniero et al., 2013; Chiappini et al., 2020) and the visual system (Romei et al., 2016; Chiappini et al., 2018), proving the effectiveness of these protocols in modulating cortico-cortical connectivity at a neurophysiological and behavioral level. More recently, cc-PAS was used to target high-order frontal areas of the human brain, allowing the study of connectivity and plasticity within complex systems crucial for cognition.

**FIGURE 1:**
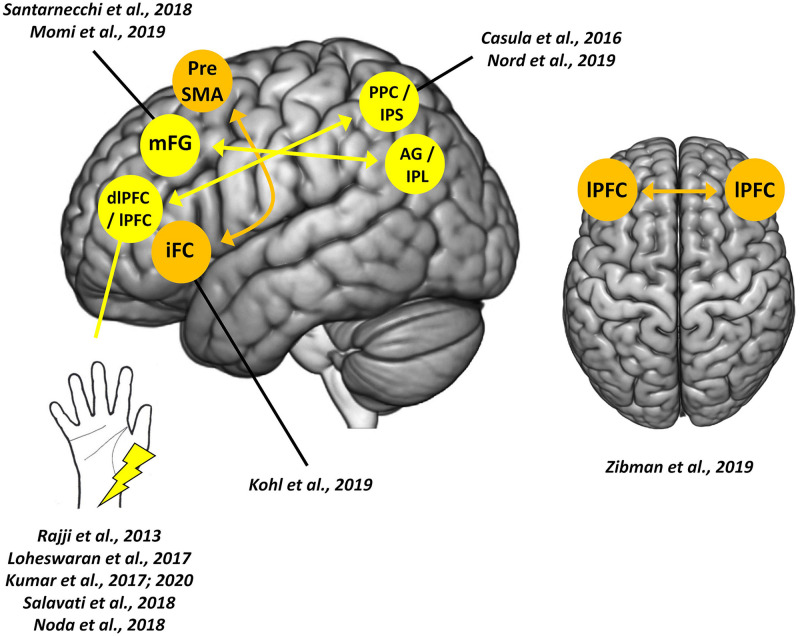
*Paired associative stimulation* (PAS) targeting frontal cortices. Colored circles indicate sites of cortical stimulations; arrows indicate the direction of the cortico-cortical connection tested. The left hemisphere is depicted only for visualization purposes and does not reflect the hemisphere stimulated in the single study or by the single protocol.

Here we provide a review of PAS protocols (both *peripheral-cortical* and *cortico-cortical*) targeting frontal networks, discussing their theoretical and clinical potentialities (see Table 1 and Figure 1; Table 1 also reported the different parameters – e.g., frequency and number of paired stimuli – exploited by the reviewed PAS).

**TABLE 1:** *Paired associative stimulation* (PAS) targeting frontal cortices.

PAS	Studies	PAS parameters	Peripheral stimulus/first (TMS) pulse	Cortical stimulation/second (TMS) pulse	Effective ISI	Plastic effects	Target cognitive function
dlPFC-PAS	Rajji et al., 2013; Kumar et al., 2017, 2020; Loheswaran et al., 2017; Noda et al., 2018; Salavati et al., 2018	180 stimuli @ 0.1 Hz (30 min)	Median-nerve electric stimulation (right hand)	Left dlPFC	25 ms	↑	Working memory
cc-PAS targeting fronto-parietal connectivity	Casula et al., 2016	100 pulses @ 0.2 Hz (8 min)	Left dlPFC	Left PPC	10 ms	↑	Sustained attention
					−10 ms	↓	
	Nord et al., 2019	100 pulses @ 0.2 Hz (8 min)	Right lPFC	Right IPS	−10 ms	↓	Decision making
	Santarnecchi et al., 2018; Momi et al., 2019	180 stimuli @ 0.2 Hz (15 min)	Left mFG	Left AG/IPL	10 ms	↑	Fluid intelligence
					−10 ms	↓	
cc-PAS targeting frontal lobes	Kohl et al., 2019	100 stimuli @ 0.2 Hz (8 min)	Right IFC	Right preSMA	4 ms	↑	Response inhibition
					−10 ms	↓	
	Zibman et al., 2019	210 stimuli @ 0.25 Hz (15 min)	Right/left lPFC	Left/right lPFC	8 ms	↑/↓	Emotional processing

*Effective protocols or replicated parameters are reported. For the ISIs, we reported the effective ones found with these protocols. **↑**: excitatory effects; **↓**: inhibitory effects.*

## Neuroplasticity Induction in Frontal Networks

In 2013, the PAS protocol was used for the first time to induce STDP in frontal areas by targeting the dorsolateral prefrontal cortex (dlPFC; Rajji et al., 2013). This *peripheral-cortical* PAS (i.e., dlPFC-PAS) repeatedly pairs median-nerve electric stimulations of the right wrist with TMS pulses over the left dlPFC. An ISI of 25 ms was deployed according to previous neurophysiological evidence that median-nerve somatosensory evoked potentials produce a negative peak in frontal areas after 25 ms, which amplitude is maximal over the electrode overlying dlPFC (i.e., F3) (Valeriani et al., 1998). Employing TMS and electroencephalography (EEG) co-registration, it was shown an enhancement of the cortical-evoked activity of dlPFC [assessed by measuring TMS-evoked potentials (TEPs)], along with a potentiation in the coupling between theta and gamma band cortical oscillations – two frequency bands related to dlPFC functioning and working memory (e.g., Canolty and Knight, 2010; Roux and Uhlhaas, 2014). Conversely, when a longer ISI was used (i.e., 100 ms), no effects were detected, proving the timing dependency of the dlPFC-PAS. Unfortunately, possible behavioral modulations on cognitive functions related to dlPFC were not assessed (Rajji et al., 2013).

The neurophysiological substrates of the dlPFC-PAS were further investigated by Salavati et al. (2018). Considering that synaptic LTP depends on glutamatergic neurotransmission and is modulated by cholinergic, dopaminergic, and GABA-ergic neurotransmission (Malenka and Bear, 2004). Salavati et al. (2018) investigate whether drugs influencing these neurotransmitters could modulate the effects of the dlPFC-PAS. Results showed that PAS after-effects (i.e., dlPFC-TEPs) are enhanced by L-DOPA and rivastigmine; these two drugs increase dopaminergic and cholinergic tone. Conversely, dextromethorphan intake, by blocking glutamatergic receptors, inhibits the protocol’s effects (Salavati et al., 2018).

Given the central role of dlPFC in reward processing and addiction pathophysiology (e.g., Loheswaran et al., 2016a, b). Loheswaran et al. (2017) took advantage of the dlPFC-PAS to investigate the effects of alcohol consumption on dlPFC neuroplasticity. Results showed that the intake of alcohol before the dlPFC-PAS impaired PAS-induced plasticity within dlPFC compared to the assumption of a placebo beverage. Furthermore, alcohol suppressed the potentiation of theta-gamma coupling (Loheswaran et al., 2017).

The protocol was also used in patients with major depressive disorder, overall suggesting lower plastic effects in this condition. The dlPFC-PAS is indeed still effective in patients with major depression. However, the magnitude of dlPFC-TEPs enhancement is lower and lacks the modulation of theta-gamma coupling, as compared to healthy conditions (Noda et al., 2018).

The dlPFC-PAS was also exploited in patients with Alzheimer’s disease (AD) to investigate their impaired frontal plasticity and its relationship with working memory deficits, known to be a dysfunctional marker of AD (Baddeley et al., 1991). Firstly, it was shown that AD presented reduced LTP-like responses to the dlPFC-PAS: in AD patients, the PAS enhancement effects on TEPs were present but significantly reduced after the protocol administration, compared to healthy controls. However, as observed in healthy subjects, even in AD patients, the protocol was able to affect the performance in a working memory task (n-back task), and the greater was the neurophysiological enhancement (TEPs), the more significant the improvement of performance (Kumar et al., 2017). In a second study by the same research group, the dlPFC-PAS was applied in AD as a treatment protocol, comprising a 2-weeks (five applications/week) of dlPFC-PAS. Exploratory results showed, after 1 day from the end of the 2-weeks treatment, neurophysiological (TEPs and theta-gamma coupling) and behavioral (n-back task) improvements, but not long-term effects (7 and 14 days after plasticity-induction), suggesting the absence of long-lasting effects and that further research is needed (Kumar et al., 2020).

Taking together, these studies exploiting the dlPFC-PAS over different populations show the usefulness of this *peripheral-cortical* protocol in modulating dlPFC functioning. Plastic effects of the protocol are indeed detectable in a cognitive function (i.e., working memory), modulated by drugs or alcohol intake, and impaired in clinical conditions affecting dlPFC functioning (e.g., major depressive disorder and AD).

A *cortico-cortical* protocol targeting homologs frontal areas was developed in 2019 (Zibman et al., 2019) to deepen the interhemispheric connectivity in frontal regions regulating emotional and motivational processing, which show asymmetrical activations in the two hemispheres (Kelley et al., 2017). This cc-PAS consisted of the repeated pairing of a TMS pulse over lPFC with ones over the homologous area of the opposite hemisphere with an ISI of 10 ms (lPFC cc-PAS). Outcomes of this protocol were assessed, behaviorally, using an emotional reactivity task and, neurophysiologically, by recording TEPs over lPFC and measuring possible asymmetries in alpha-band power, a marker associated with emotional processing (e.g., Allen et al., 2004). Results showed that lPFC cc-PAS effects depended on the stimulation direction: left-to-right prefrontal stimulation increased the attentional bias in the emotional reactivity task and led to a shift of alpha-band power toward the right hemisphere suggesting the induction of depressive effects in the lPFC after the protocol. Conversely, right-to-left hemisphere cc-PAS decreased attentional bias and led to a shift in alpha power in the left hemisphere. Furthermore, both cc-PAS increased interhemispheric signal propagation in the direction of the paired stimulations. To sum up, lPFC cc-PAS successfully modulated emotional processing, changing the balance of hemispheric activation in the stimulated frontal cortices (Zibman et al., 2019).

Kohl et al. (2019) introduced a variant of cc-PAS targeting the frontostriatal network by repeatedly pairing TMS pulses over the right inferior frontal cortex (iFC) with TMS pulses over the ipsilateral pre-supplementary motor area (pre-SMA), and vice versa (i.e., iFC-preSMA and preSMA-iFC PAS, respectively); two ISIs of 4 or 10 ms were tested. The 4 ms ISI would be too short to directly target cortico-cortical interaction, most likely recruiting a cortical-subthalamic pathway. On the contrary, 10 ms-ISI was used to assess whether plasticity might be directly induced in the cortico-cortical pathway connecting iFC and preSMA. A classic stop-signal task was used to assess PAS effects on response inhibition, while a delay discounting paradigm (i.e., a monetary choice questionnaire) was used as a control condition. Results showed that the effects of frontal cc-PAS in the stop-signal task varied as a function of participants’ age: younger individuals showed a more significant impairment following preSMA-iFC PAS with the ISI of 10 ms; older individuals showed improvements after iFC-preSMA PAS with 4 ms-ISI. Performance at the delay discounting paradigm was not modulated by any of the cc-PAS protocols used. These results suggested that plasticity-induction within the response inhibition network by cc-PAS might influence both cortico-subcortical and cortico-cortical communication (Kohl et al., 2019).

## Neuroplasticity Induction in Fronto-Parietal Networks

The first cc-PAS targeting a fronto-parietal cross-cortical pathway was introduced by Casula et al. (2016) and consisted of the repeated pairing of TMS pulses over left dlPFC with ones over the ipsilateral posterior parietal cortex (PPC). Two versions of the protocol were tested, varying the order of the paired stimulations: in the first with the first pulse delivered over dlPFC and the second one over PPC (fronto-parietal PAS); in the other, the order was reversed (i.e., parieto-frontal PAS). Both versions exploited an ISI of 10 ms, according to the conduction time of the parieto-frontal pathway (Koch et al., 2013). As assessed through EEG-TMS co-registration, both protocols were effective in inducing STDP-like effects at TEPs level, with a modulation resembling the so-called *anti-Hebbian* plasticity (Koch et al., 2013): indeed, LTP-like effects were found in dlPFC responses when its activation preceded the TMS pulse over PPC (fronto-parietal PAS), while LTD-like effects emerged when the PPC activation preceded the dlPFC one (parieto-frontal PAS). No effects were found for TEPs recorded over PPC. Besides, bidirectional changes in high-frequency oscillatory activity of dlPFC emerged from time/frequency-domain analysis: the fronto-parietal cc-PAS enhanced oscillatory activity in beta and gamma bands, while parieto-frontal cc-PAS decreased it, in line with previous studies relating modifications in high-frequency cortical oscillation to STDP (e.g., Azouz and Gray, 2003).

Santarnecchi et al. (2018) investigated the functional connectivity underpinnings of fronto-parietal cc-PAS using fMRI and administering a sustained attention task. The targeted frontal and parietal sites were selected according to preliminary neuroimaging data on key cortical nodes of the “task-positive” [i.e., on average, middle frontal gyrus (mFG)] and the “default mode” [i.e., angular gyrus (AG)] networks. After the administration of the parieto-frontal PAS, the network-to-network connectivity at rest between AG and mFG tended to increase. An increased blood oxygenation level-dependent (BOLD) response was found in prefrontal areas during the attentional task. Conversely, after fronto-parietal PAS, an increased BOLD response was found in parietal regions (Santarnecchi et al., 2018).

Nord et al. (2019) tested the fronto-parietal cc-PAS effects on decision-making by targeting the right lateral prefrontal cortex (lPFC) and the right intraparietal sulcus (IPS). The effects of this fronto-parietal protocol, and its reversed version (i.e., parieto-frontal PAS), were assessed using a 2-step reinforcement learning task (used to measure two different decision-making strategies, i.e., habitual and goal-directed) and a working memory task (i.e., orientation delayed-estimation task). The authors found that only parieto-frontal PAS effectively shifted decision-making from a habitual to a more goal-directed strategy. In contrast, no effects were found in working memory after both PAS protocols (Nord et al., 2019).

In the same year, Momi et al. (2019) tested cc-PAS efficacy on *fluid* intelligence, the ability to organize, filter, and extrapolate new information (Gray et al., 2003). The stimulation protocol targeted two critical nodes of the network putatively involved in *fluid* intelligence: the mFG and the inferior parietal lobule (IPL); both associative directions were tested (fronto-parietal and parieto-frontal PAS). Performance at the Sandia matrices, an abstract reasoning task that includes both logical and relational trials, represented the behavioral outcome (Matzen et al., 2010). Results showed enhanced accuracy in the relational trials of the Sandia matrices after fronto-parietal PAS. In contrast, parieto-frontal PAS enhanced the logical ones, suggesting the induction of associative plasticity according to the cortico-cortical direction of the protocol stimulations and the conditioned area’s role in the *fluid* intelligence network. These effects were specific: a letter go-no-go and a visual search task (two tasks implicating a slightly different cortical network than the Sandia matrices) were used, but cc-PAS did not lead to any modulation. Finally, when the two paired stimulations were delivered simultaneously (i.e., ISI of 0 ms), or if TMS was delivered only over mFG, no effects were found, proving the timing-dependency of the protocol and the importance of its “associative” nature to modulate *fluid* intelligence (Momi et al., 2019).

## Discussion

The frontal lobe is responsible for a vast range of high-order functions such as memory, prediction, language, motivation, social and emotional processing (e.g., Aron et al., 2004; Alvarez and Emory, 2006; Amodio and Frith, 2006; Blumenfeld and Ranganath, 2007; Badre and Nee, 2018). Frontal cortices act as a sort of “elaboration hub” where the integration of information – and thus, connectivity – with other brain regions is crucial for optimal functioning (Stuss and Knight, 2013). Given their central role, exploring and testing novel methods to investigate their functional properties, even affecting them, is crucial for cognitive and system neuroscience, with potential translational impacts in neurorehabilitation. The use of PAS to study and assess forms of plasticity-induction in frontal areas and networks has also allowed defining their timing-dependent constraints. The importance of communication between nodes of a cortical network is crucial for the functioning of complex systems like the ones subtended to cognitive and executive processes (van den Heuvel and Hulshoff Pol, 2010; Friston et al., 2011). One potential critical issue of targeting a cortical region involved in a high-order cerebral network is that this area/region would not be the only one selectively implicated in the targeted cognitive function/process. PAS can (partially) overcome this limitation by activating two specific network nodes within a precise temporal window. Of relevance, the after-effects induced by frontal PAS protocols are also detectable at the behavioral level, even if not always with a clear correspondence to the neurophysiological findings. Nevertheless, many theoretical questions remain unexplored from the protocols described here and future studies are required to confirm – and replicate – the effectiveness of all frontal PAS. Considering the newness of all the protocols described, future studies should be focused on deepening the neurophysiological bases of these protocols by better characterizing the implicated cortical (or sub-cortical) pathway as well by uncovering the contribution of stimulation parameters (e.g., ISIs, stimulation intensity, number of paired stimuli) supporting PAS effectiveness.

Some critical consideration has to be made from the results presented in this mini-review. Firstly, none of the cc-PAS studies described here introduces a preliminary investigation to assess the precise conduction time of the stimulated cortico-cortical pathway, merely exploiting timings taken from previous literature, even regardless of the target area (e.g., Casula et al., 2016; Zibman et al., 2019). However, these protocols were still effective. An intriguing question arises: is it possible that in PAS targeting high-order functions, the use of an ISI resembling the precise conduction time of the stimulated pathway, a key characteristic of PAS targeting the motor system, is not necessary? We suggest that the answer is not so straight-forward, and further research is needed. Indeed, as already proved for PAS targeting sensory-motor networks (e.g., Koch et al., 2013; Chiappini et al., 2018, 2020; Zazio et al., 2019; Maddaluno et al., 2020), different confounding factors, which are likely to be more involved in protocols targeting complex networks – e.g., bidirectional interplay within and between cortical regions, activation state of the stimulated network, participants’ attention and expectancy during the protocol’s administration – can influence the effectiveness of PAS’ ISIs and, in a broader perspective, the effectiveness of the PAS. For instance, due to the augmented complexity of the cortico-cortical pathways activated by cc-PAS, the temporal windows (i.e., range of effective ISIs) to induce excitatory/inhibitory changes may be wider and less strict than in PAS targeting primary sensory systems (e.g., Wolters et al., 2003, 2005). However, for PAS targeting frontal cortices, this remains speculation, and future studies should deepen this crucial aspect.

Another critical characteristic of Hebbian associative plasticity is its long-lasting nature, which cannot be entirely confirmed from the present results. Studies exploiting PAS over the motor system showed that the induced plasticity might last, at least, as the time of protocol administration (for a review, see: Suppa et al., 2017). Hence, in most of the works here described, PAS after-effects are assessed immediately after the end of the protocol. However, it cannot be *a priori* excluded that the induced plasticity may last even longer. For instance, Kumar et al. (2020) found that plastic effects of the dlPFC-PAS are still detectable 1 day after the end of a treatment where the protocol is administered over a 2-weeks period for 5 days/week.

Another undisclosed methodological issue that deserves further investigation is related to the influence of TMS intensity (either of the first and the second pulse) on the effectiveness of PAS, notwithstanding the recent evidence of its crucial role, especially in paired-pulse TMS paradigms or when frontal TEPs are measured (e.g., Bäumer et al., 2009; Zanon et al., 2018; Rawji et al., 2021). For future studies aiming to develop novel frontal PAS, we suggest to carefully consider these methodological works on the importance of TMS intensity, thus to select the better parameters to stimulate (and modulate) the target cortical areas.

The considerations made so far allow us to highlight another critical methodological note: if the reader aims to use one of these protocols, we suggest adopting – at least in one of the experimental conditions – the same effective parameters exploited in the studies here presented (see Table 1). Indeed, even slight modifications (such as a minor number of stimuli or a different stimulation frequency) can potentially influence the protocol’s effectiveness, as already highlighted from *peripheral-cortical* PAS targeting sensory and motor systems (for a review, see: Wischnewski and Schutter, 2016; Suppa et al., 2017).

However, from the evidence obtained so far – and the open questions discussed – frontal PAS’ future seems bright. For instance, by engaging relevant cortical networks and manipulating temporal dynamics supporting specific cognitive and executive functions (like sustained attention or fluid intelligence; e.g., Santarnecchi et al., 2018; Momi et al., 2019), frontal PAS can be exploited to validate cognitive models causally. Certainly, adopting complementary techniques like fMRI or EEG would be essential for investigating PAS effects in complex frontal networks.

The clinical population suffering from dysfunctions in frontal networks and related connectivity would undoubtedly represent a fertile ground for these protocols. As seen here, the first preliminary attempts to exploit these protocols in the clinical population were made on AD patients (Kumar et al., 2017, 2020). Nevertheless, we suggest that this investigation can be further deepen using cc-PAS targeting fronto-parietal networks (e.g., Casula et al., 2016; Santarnecchi et al., 2018), like the default mode network, which is known to be dysfunctional in AD (e.g., Jones et al., 2011; Agosta et al., 2012; Bagattini et al., 2019). The use of these protocols can also be extended to other forms of frontal dementia or to mild cognitive impairment to study the plastic potential of neurodegenerative diseases and, possibly, the neurophysiological prognostic factors likely mediated by dysregulations in cortical connectivity or atrophy (e.g., Nardone et al., 2014; Rajji, 2019; Di Lorenzo et al., 2020). Furthermore, PAS seems to be well tolerated in the elderly, likely due to the lower frequency of stimulation than other repetitive TMS protocols (e.g., Kumar et al., 2020).

Besides neurodegenerative disorders, the investigation and the treatment of psychiatric conditions mediated by dysfunctions in frontal networks, such as schizophrenia, depression, or addiction disorders, would also benefit from these novel protocols. For instance, considering depression or addiction disorders, the evidence that classical rTMS protocols are successful in such clinical conditions (for a review, see: Padberg and George, 2009; Diana et al., 2017) suggests that frontal PAS can be used with promising results too (Noda et al., 2018). Furthermore, the effectiveness of cc-PAS in determining significant changes also in the oscillatory activity of the human brain contributes to validate these protocols further and opens the possibility of applying them in the neurorehabilitation field. High-frequency oscillatory activity has been connected to several cognitive functions (e.g., Santarnecchi et al., 2013; Picazio et al., 2014; Casula et al., 2016). The possibility to selectively manipulate the functional connectivity with a high temporal and topographical specificity – key advantages of PAS protocols – may promote circuit reorganization in patients with an imbalance in the oscillatory activity of a specific cerebral network and pave the way for novel therapeutic tools (Veniero et al., 2013; Casula et al., 2016).

In conclusion, recent literature highlights how PAS protocols are valuable tools for studying timing-dependent plasticity outside sensorimotor networks, allowing to induce it in frontal cortices and related networks. Future studies are needed to replicate and deepen the results found with the protocols described in the present review. Still, this investigation seems worthy: this would shed better light on the plastic properties of the human brain’s high-order cognitive networks.

## Author Contributions

GG conceived the review. GG and CR searched the relevant literature and wrote the first draft of the manuscript. All authors equally contributed to the writing and editing of the final version of the manuscript.

## Conflict of Interest

The authors declare that the research was conducted in the absence of any commercial or financial relationships that could be construed as a potential conflict of interest.
